# Engagement of people with lived experience of dementia advisory group and cross-cutting program: reflections on the first year

**DOI:** 10.1186/s40900-022-00359-5

**Published:** 2022-06-25

**Authors:** Ellen Snowball, Rosette Fernandez Loughlin, Heather Eagleson, Karen Myers Barnett, Emily McLellan, Denis O’Connor, Catherine Kelly, Christine Thelker, Katherine S. McGilton, Jennifer Bethell

**Affiliations:** 1 KITE Research Institute, Toronto Rehabilitation Institute, University Health Network, 550 University Avenue, Toronto, ON M5G 2A2 Canada; 2 Engagement of People with Lived Experience of Dementia Advisory Group and Cross-Cutting Program, Canadian Consortium on Neurodegeneration in Aging, Montreal, Canada; 3 Lawrence S. Bloomberg Faculty of Nursing, University of Toronto, 155 College Street, Toronto, ON M5T 1P8 Canada; 4 Institute of Health Policy, Management & Evaluation, Dalla Lana School of Public Health, University of Toronto, 155 College Street, Toronto, ON M5T 1P8 Canada

**Keywords:** Patient and public engagement, Dementia, Aging, Health research, Lived experience of dementia, Multi-stakeholder, Patient leadership, Patient-oriented research, Advisory group, Engagement in research

## Abstract

**Background:**

The objective of this paper is to describe the activities, challenges and mitigation strategies, lessons learned and reflections on the importance of engagement from the first year of the Canadian Consortium on Neurodegeneration in Aging (CCNA) Engagement of People with Lived Experience of Dementia (EPLED) Advisory Group and cross-cutting program. EPLED was created to support persons with dementia and care partners to be actively involved in the CCNA research process.

**Main body:**

The Advisory Group was formed to work with CCNA researchers and programs to develop new ways to further collaborate and advance the methods of patient engagement in research on dementia. A role profile and recruitment poster were developed and, after interviews, 17 people were invited to join the Advisory Group. We planned three online EPLED meetings to take place between July–August of 2020, with one in-person meeting to be held in Canada. Due to COVID-19, we moved all of these meetings online. In the first year, EPLED and the Advisory Group met seven times formally, four times informally, developed a website, engaged with CCNA research projects, participated in CCNA “Central” activities and formulated an evaluation plan. For researchers and people with lived experience of dementia, motivations for patient engagement included challenging stigma, making meaning from their experience (such as building relationships and having their voices heard) and contributing to research. Common challenges to engagement were related to navigating the impact of COVID-19, such as difficulty in getting to know each other and technical issues with video-conference software. We learned that developing trusting relationships, providing education, offering support, being flexible and acknowledging tensions between research, practice and lived experience, were vital to the success of the Advisory Group.

**Conclusion:**

The first year of the EPLED Advisory Group demonstrated the potential contributions of people with lived experience of dementia as partners in research. Building these collaborations with individuals and communities—people living with dementia, care partners, researchers and research institutions—has the potential for positive impact across these groups and, ultimately, improve the lives of people living with dementia and their care partners.

## Plain English summary

In this paper, we describe the development of the Canadian Consortium of Neurodegeneration in Aging (CCNA) Engagement of People with Lived Experience of Dementia (EPLED) cross-cutting program and the first year of the EPLED Advisory Group. EPLED was created to build opportunities for patient engagement in the CCNA’s research on dementia. People living with dementia and current/former friends, family and care partners were recruited from across Canada to join the Advisory Group. In the first year, the Advisory Group met seven times formally, four times informally, developed a website, engaged with CCNA research projects, participated in CCNA “Central” activities and formulated an evaluation plan. Challenges included the impacts of the COVID-19 pandemic, online meetings, and differing expectations and priorities. Lessons learned and reflections on the importance of engagement are discussed in the context of research on dementia and from the perspectives of researchers and Advisory Group members.

## Background

The term “dementia” does not refer to one specific disease but is an umbrella term used to describe a set of symptoms related to neurodegenerative illnesses, or diseases, affecting the brain. Types of dementia include Alzheimer’s disease, mixed dementia, vascular dementia, dementia with Lewy bodies, and often share common symptoms such as memory loss (both short-term and long-term), difficulties in thinking, problem-solving and language, and changes in mood and behaviour. Dementia is progressive, meaning that as brain cells become damaged by the disease and eventually die, symptoms become worse and can eventually become severe enough to affect the person's ability to perform everyday activities [1]. According to the Alzheimer’s Society of Canada, over half a million Canadians are living with Alzheimer’s disease or another form of dementia and this number will nearly double by 2030 [2, 3]. To address such developments, in 2014, over 350 clinicians and researchers came together to form the Canadian Consortium on Neurodegeneration in Aging (CCNA) with the aim of advancing research on neurodegenerative diseases [4]. CCNA researchers are organized into 19 teams that work in the areas of prevention, treatment and quality of life. These teams are supported by cross-cutting programs, which help with various aspects of their research, including Training and Capacity Building; Knowledge Translation and Exchange; Ethical, Legal, and Social Issues; and Women, Sex, Gender and Dementia [4]. The CCNA is funded by Canadian Institutes of Health Research (CIHR) and partner organizations. The CCNA is currently in its second 5-year funding cycle; Phase I of the CCNA spanned 2014 to 2019 and Phase II began in 2019.

In recent years, patient (and public) engagement [5, 6] or involvement [7] in research has been recommended by funding agencies in the United States, Canada, and the United Kingdom [8]. It has been argued to lead to better quality research with greater impact. It is also advocated as a human right, whereby the public, and particularly people impacted by the research, can have a say in publicly funded research [9]. The Engagement of People with Lived Experience of Dementia (EPLED) is a new cross-cutting program developed in CCNA Phase II, supported by the Alzheimer Society of Canada as part of its commitment to the CCNA. The objectives of EPLED are to: (1) Support persons with dementia and care partners to be actively involved in the CCNA research process; (2) Work with CCNA research teams, cross-cutting programs and partners to develop novel mechanisms and formats to further this collaboration; and to (3) Advance the methods of patient engagement in research, including specifically for persons with dementia and care partners, by embedding evaluation processes that will measure the impact of these CCNA initiatives. EPLED is co-led by two academic researchers (JB and KMcG) and managed by a research analyst (ES).

From September to December 2019, EPLED conducted an online, anonymous survey of CCNA researchers to describe their knowledge, attitudes, and activities related to patient engagement in dementia research; 93% of respondents agreed that people with lived experience could contribute to research—and many were already incorporating engagement activities in their work [10]. Many respondents also indicated that barriers to incorporating engagement in their research were related to; resources (such as time and money) and to finding people with lived experience who were interested and knowledgeable about being engaged in research [10]. Suggestions for enabling engagement within CCNA included developing ways to connect researchers and people with lived experience of dementia [10]. These findings motivated the development of an Advisory Group composed of individuals with lived experience of dementia who would work with CCNA researchers—not as study subjects but as collaborators in research. Advisory Group members would be supported to participate in research activities related to governance, priority setting, conducting research and knowledge translation.

The aim of this paper is to describe the activities, challenges and mitigation strategies, lessons learned and reflections on the importance of engagement from the first year of the EPLED Advisory Group—from the perspectives of researchers and Advisory Group members.

## Activities

In the first year, EPLED recruited the Advisory Group and they participated in a number of activities; they met seven times formally, four times informally, developed a website, engaged with CCNA research and activities and formulated an evaluation plan:

### Recruitment

In January 2020, EPLED began the process of recruitment for the Advisory Group with a lay-language poster and application form that was shared on the CCNA website. Anyone with lived experience—people living with dementia, friends, family members, and caregivers/care partners—were encouraged to apply. The poster and application form also noted that meetings would be carried out in English (Canada’s two official languages are English and French). The application form asked those with diverse perspectives, such as young carers, members from the 2SLGBTQAI + community and those with Indigenous and racialized backgrounds, if they would be willing to share their perspectives through the Advisory Group. The EPLED poster and application form can be found at: https://www.epled.ca/en/joining.

The posting was circulated by Alzheimer’s Societies in Canada as well as through Strategy for Patient Oriented Research (SPOR) support units in provinces across Canada. Along with the poster, a lay-language role profile was developed detailing the goals of the Advisory Group membership. Following SPOR guidelines, EPLED also specified remuneration for participation and expenses incurred [11]. Key responsibilities were described as; providing input on research questions and aspects of study design (e.g., ensuring methods are acceptable and equitable for potential research participants), reviewing study documents (e.g., ensuring language and content of consent forms and data collection methods are appropriate and accessible), analyzing and interpreting data (e.g., contextualizing results by providing perspectives of lived experience), and assisting with knowledge translation (e.g., helping to ensure study results are communicated in a way that is accessible and meaningful). The EPLED role profile can be found at: https://www.epled.ca/en/materials.

Recruitment was closed in March 2020, with 62 applications from people with lived experience of dementia from across Canada. The applications were reviewed by JB, ES and KM and applicants were selected for interview based on their responses, including their experience with dementia (all those who identified as a person living with dementia were invited to an interview), region within Canada and self-reported willingness to contribute perspectives of diversity (e.g., age, gender, racialized perspectives, etc.). In April and May 2020, 27 applicants were contacted for a short interview via videoconference. A list of 10 standardized questions were created by JB and ES, covering topics such as related volunteer and work experience, specific interests in participating in the Advisory Group as well as anticipated contributions and personal benefits, and suggestions for the Advisory Group role profile. Applicants were also asked about current role responsibilities, communication preferences, and accessibility needs and support as well as if they had questions for EPLED. These questions were shared with the applicants one week in advance of their interview appointment. After considering information obtained in the applications and interviews, and to maximize diversity, 17 individuals from across Canada were invited to join the Advisory Group.

### Advisory group membership

17 members were invited to join the Advisory Group; five (29%) were people living with dementia and 12 (71%) were current or former care-partners or caregivers to a person with dementia (including five spouses and seven adult children). Overall, women (n = 10; 59%) outnumbered men (n = 7; 41%) and most were from Ontario (n = 7; 41%), then British Columbia (n = 4; 24%), Alberta (n = 3; 18%), Newfoundland (n = 2; 12%) and Nova Scotia (n = 1; 6%). More information about Advisory Group members can be found at www.epled.ca/en/advisory-members.

### Meetings

The first meeting was initially planned to take place in-person to help build relationships within EPLED and the Advisory Group, with the remainder of the meetings to be held online. However, like many others, all meetings were moved online because of the coronavirus (COVID-19) pandemic. Four online (phone or videoconference) meetings were held over July and August, 2020. To prepare for online meetings in advance, Advisory Group members were emailed meeting agendas, online calendar invites and instructions on how to connect using Microsoft Teams. 1-h pre-meeting reminders and technical support were provided on an ongoing basis.

The first meeting, on July 13th 2020, focused on welcoming and introducing the members to each other and reviewing objectives for the Advisory Group. Plans for upcoming meetings and training sessions, compensation, and terms of reference were also discussed. At the second meeting on July 27th, 2020, a guest from the BC SUPPORT (Support for People & Patient-Oriented Research & Trials) Unit delivered the “Module 2: Fundamentals of Health Research in Canada” curriculum developed by CIHR [12]. This curriculum was designed to give participants a foundational understanding of health research; it articulated learning outcomes related to the purpose of health research, those involved (including researchers, research funders, study subjects), the diversity of topics and approaches used in health research, ethical considerations, peer review, knowledge translation and exchange. Advisory Group members received a certificate of completion from CIHR. At the third meeting on August 10th and at the fourth meeting on 28th, 2020, Dr. Howard Chertkow, Scientific Director of the CCNA, welcomed the Advisory Group to the CCNA, provided an overview and answered any questions. In August 2020, a draft term of reference was created by JB, ES and KMcG based on resources from Health Quality Ontario and Alberta SPOR SUPPORT Unit [13, 14]. The draft was circulated to the Advisory Group by email for feedback and any revisions to the document were discussed during subsequent meetings. The EPLED terms of reference articulates membership of no more than 20 people, all with lived experience of dementia (i.e., people living with dementia and/or friends, family and caregivers/care partners). Advisory Group role responsibilities included that each member serve a 1-year term, with opportunities for continued membership; members are to inform the EPLED program staff if they can no longer participate; members are to inform the program leads or staff liaison if they will miss a meeting or need additional support; and participation in specific research projects and other CCNA-related activities is at the discretion of individual members with no obligation to participate in any particular initiative.

EPLED had initially planned for Advisory Group meetings to take place quarterly, However, in January 2021, EPLED introduced monthly, informal meetings. The purpose of the first monthly meetings was for Advisory Group members to get to know one another. Over time, these meetings evolved into opportunities for EPLED leads and Advisory Group members as well as CCNA researchers and other guests to address specific topics of interest.

In May and June of 2021, the EPLED team conducted one-on-one meetings with each of the Advisory Group members to discuss any concerns or questions about the Advisory Group, clarify role expectations, identify areas for improvement and inquire about interest in continuing for another year. On July 13th, 2021, EPLED held a special session to celebrate the Advisory Group’s 1-year anniversary milestone.

### Website and communications

In September of 2020, EPLED and Advisory Group members co-created a website (www.epled.ca), which, using relevant literature and guidelines, focused its design on being accessible for people living with dementia. Existing literature on accessible digital and print design were consulted, including resources compiled from guidelines, academic journals, and recommendations [15–20]. EPLED incorporated the following design choices: a simple template with clear headings and links, icons and images to help promote easy navigation, larger font sizes and a Sans Serif font, accessible colour palettes, clear and concise content by measuring “readability”, such as use of plain language, free from jargon or words that are hard to understand [15–20]. Design resources and examples were shared with the Advisory Group, along with a draft website. Advisory Group feedback was incorporated into the website design and revisions were discussed on an ongoing basis.

The EPLED website provides information about the Advisory Group members and materials (e.g., role profile, terms of reference), an online form for CCNA researchers who are interested in engaging the Advisory Group, engagement resources (e.g., references and tips for researchers), and other information, including website design resources.

Internally, EPLED also designed a bi-weekly email newsletter informing Advisory Group members of relevant online events, research papers, upcoming meetings and news from within the Advisory Group.

### Engagement with CCNA research and activities

In October 2020, to build awareness of the Advisory Group within CCNA, EPLED presented a poster and hosted a workshop for CCNA’s 2020 “Partners Forum and Science Days” (see: https://ccna-ccnv.ca/pfsd-2020/), and contributed to the CCNA’s “La Capsule” newsletter (see: https://ccna-ccnv.ca/la-capsule-ccnas-monthly-newsletter/ October 2020 Edition).

Within the first year, Advisory Group members became involved in four CCNA research projects with 3 CCNA teams: Team 5 Diet and Prevention “Brain Health Food Guide”, Team 16 Driving and Dementia “Equipping Healthcare Providers with the Knowledge & Skills to Support Driving Cessation”, Team 19 Integrating Dementia Patient Care into the Health Care System “Understanding and Improving the Care of Older Adults Living with Dementia Across Four Canadian Provinces during the COVID-19 Pandemic: A Mixed-Methods Study to Inform Policy and Practices”) and “Co-designing Dementia Diagnosis and Post-Diagnostic Care”. It is beyond the scope of this paper to describe specific activities within each of these projects, however, they included priority setting (e.g., prioritizing research questions [21]) and conducting research and knowledge translation activities (e.g., co-designing and participating in a website and campaign aimed to improve the dementia diagnosis and post-diagnosis experience [22]). EPLED and Advisory Group members also collaborated with CCNA “Central” activities, including the COVID-19 and Dementia Taskforce, [23, 24] as well as with other cross-cutting programs, including a resource list and video for the Training and Capacity Building program website [25], Knowledge Translation & Exchange (e.g., vascular cognitive impairment journey map, summary of Aducanumab statement [26] and Annual Science Days Planning Committee [27]) and a webinar with the Stigma & Social Inclusion group [28].

### Evaluation

EPLED worked with the Advisory Group to develop an approach to evaluate EPLED activities in order to improve the program and plan future activities. Evaluation would include capturing quantitative data on Advisory Group activities (e.g., number and nature of projects) as well as Advisory Group and CCNA perspectives on the engagement activity. Existing resources [29–31] were used to identify key domains and Advisory Group members were invited to share their perspectives on the most relevant questions, including by voting on existing questions or suggesting their own. An existing tool, the Patient Engagement in Research Scale (PEIRS-22) [32], was subsequently located and Advisory Group responses were mapped to this validated tool. The PEIRS-22 was selected, but with two additional questions, from the full (37-item) PEIRS and free-text questions. EPLED also developed a short questionnaire for CCNA researchers to evaluate their experience working with Advisory Group members, including questions related to timing, objectives, engagement, collaboration and impact. Both questionnaires were transferred to Google Forms for online data collection. These data will be reported and presented internally and externally as part of EPLED’s year 2 activities.
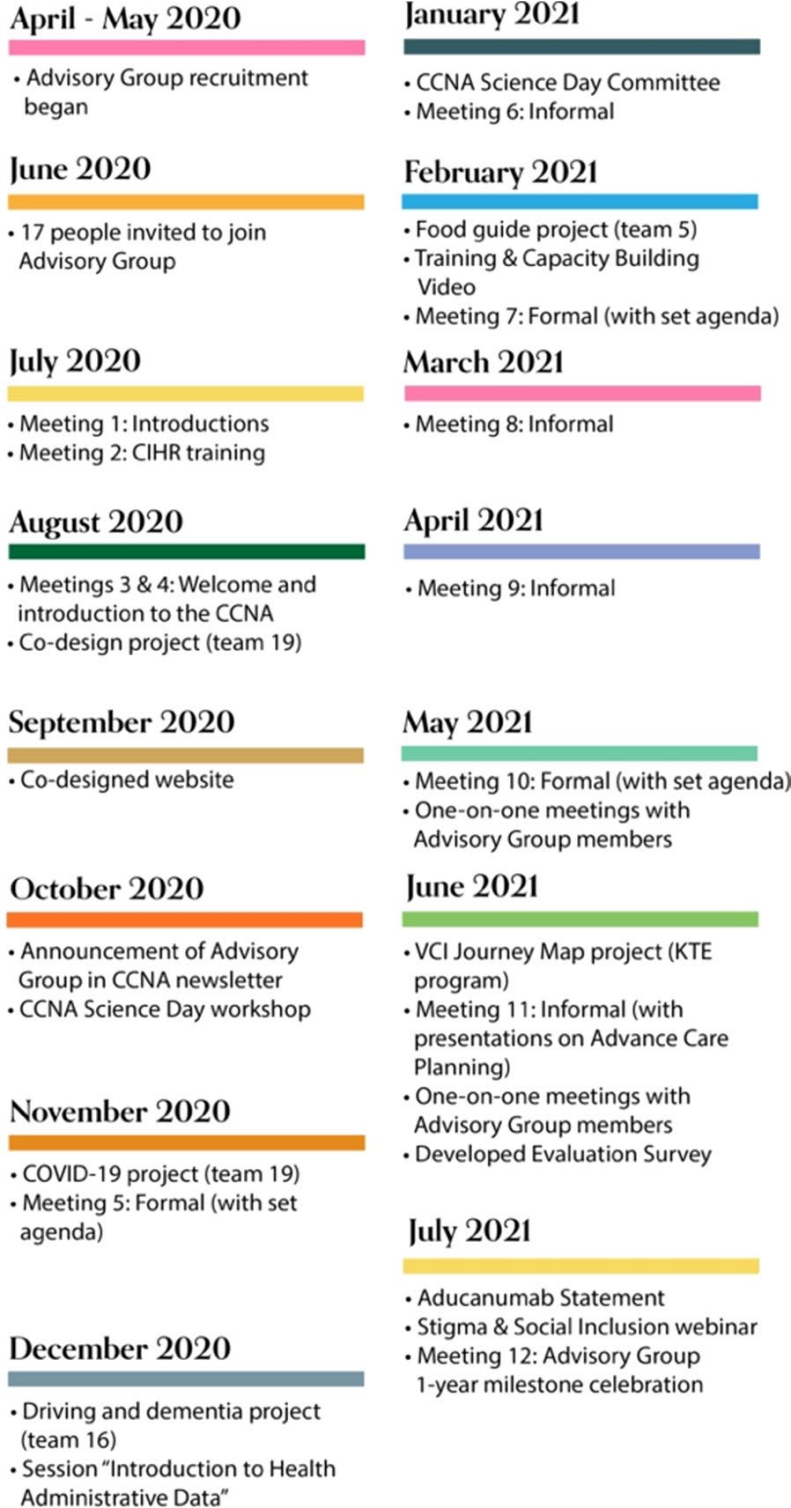


## Challenges and mitigation strategies

During its first year, the EPLED Advisory Group experienced a few challenges along the way, including:

### COVID-19

When COVID-19 was declared a global pandemic in March 2020, our plans for one in-person meeting per year were cancelled. Unfortunately, members could not meet in-person and all meetings were moved online, which presented specific challenges for EPLED and the Advisory Group. First, the planned training module, Fundamentals of Heath Research in Canada, was designed to be delivered in-person, over 2–3 h. EPLED worked with the BC SUPPORT Unit who adapted this training to a shortened online format. Second, without the opportunity to meet in-person, it took longer to develop relationships within the Advisory Group and with EPLED. To address this, EPLED added informal monthly meetings with no set agenda. These meetings evolved into time that could be used to address specific topics of interest or concern, where EPLED could invite Advisory Group members, CCNA researchers, and other guests to present and partake in the conversation. COVID-19 also presented bigger and more personal challenges to the Advisory Group. Many Advisory Group members were isolated from their family and some experienced a loss of a loved one. Some resided in long-term care homes, where residents experienced disproportionately high morbidity and mortality [33]. During the pandemic, from March 1st 2020 to February 15th 2021, more than 2,500 long-term care and retirement homes across Canada experienced a COVID-19 outbreak, leading to the deaths of over 14,000 residents [34]. EPLED endeavored to support Advisory Group members but also encouraged them to prioritize their own health and well-being and that of their families. Some Advisory Group members discussed that, despite the various challenges they experienced during the COVID-19 pandemic, they took strength from the relationships and projects they developed through their work with the Advisory Group.

### Online meetings

In addition to one in-person meeting per year, the role profile for Advisory Group members articulated three online meetings per year. Challenges to holding online meetings included scheduling meetings with Advisory Group members who were in time zones across Canada (from Newfoundland to British Columbia, 4.5 h difference), communicating meetings details and selecting an online videoconference platform that accommodated member preferences and devices (e.g., phone, tablet or computer). To address these challenges, EPLED coordinated meetings by polling Advisory Group members with potential meeting dates and times and a 2021 meeting calendar was set and communicated in January 2021. EPLED communicated meeting schedules in multiple formats, namely, by email, with online calendar invitations, a shared calendar on the EPLED website, an “upcoming dates” section in the biweekly email newsletter and 1-h pre-meeting reminders. EPLED also provided technical support, over phone and email, to help address technical issues. After polling the Advisory Group, EPLED also switched videoconference platforms to one that was more familiar to Advisory Group members. Ultimately, EPLED endeavored to make meetings more dementia-friendly and more accessible to all members by making efforts to ensure meetings were scheduled, communicated and conducted in a manner that was conducive to Advisory Group members’ participation.

### Differing expectations and priorities

EPLED represents a collaboration between researchers and people with lived experience. While aligned in their goals of improving health and quality of life for people living with dementia and their families, the process and speed through which to achieve these goals was at times a point of discussion. Advisory Group members sometimes discussed opportunities for advocacy as well as the more immediate need for public and care provider education to improve practice whereas EPLED researchers articulated longer term academic goals (e.g., grants and publications). The role profile that was initially developed for Advisory Group recruitment was used to clarify activities in research. Opportunities for Advisory Group members to support knowledge translation activities were also pursued through collaborations with the CCNA Knowledge Translation & Exchange program.

## Lessons learned

Through the first year, the EPLED Advisory Group members have discussed some important lessons for engaging in CCNA research which were discussed during group and one-on-one meetings and in collaborating on this paper. Some common themes were:

### Develop trusting relationships

Establishing strong working relationships, upheld by mutual respect, trust and communication, were vital for EPLED and the Advisory Group. Valuing different perspectives, giving equal opportunity for feedback, and providing support and clarity on roles were essential to building reciprocal relationships. It took some time for these relationships to develop. This collective learning process allowed us an opportunity to understand the unique and complex challenges members faced and what we could do to better support each other. We found it helpful to host informal check-ins for members and EPLED to catch up, socialize and support each other. We learned the significance of making sure there were plenty of opportunities for Advisory Group members to share their insights. As we got to know each other, we witnessed the collective identity of the Advisory Group beginning to take shape; confidence in speaking up grew and trusting relationships were formed among members.

### Provide education and support

There was diversity within the Advisory Group with respect to experience with research; some had been involved in other research projects in similar capacities and some had previous direct research experience. Others found their roles as co-researchers to be confusing. EPLED provided initial training through the CIHR “Fundamentals of Health Research in Canada” curriculum. Additionally, the year provided ongoing opportunities to educate members about research processes (e.g., applying for grants, research ethics board roles, academic publishing, etc.) as well as the prolonged timelines often involved in research.

### Be flexible

Advisory Group members came from unique geographical locations, backgrounds and experiences. Members faced potential barriers to involvement with the EPLED Advisory Group, such as managing scheduled meetings with a care partner, work, or family responsibilities, difficulties with technology or volume of information. To support Advisory Group members, EPLED developed a flexible approach; attending meetings and participating in CCNA research projects was always at the discretion of the Advisory Group member. An advantage of hosting meetings online was that it was easier to invite guest speakers from all across Canada, and reschedule meetings if needed. Advisory Group members felt that they were more productive as a result of meeting online, in part because of spending more time at home during the pandemic. Still, members’ circumstances, interests and availability change over time, including as a result of changes related to dementia. There was open communication between EPLED and Advisory Group members about these changing needs and expectations.

### Acknowledge tensions and imbalances

In EPLED, both researchers and Advisory Group members share a common goal: to improve the lives of those affected by dementia. Establishing shared understanding on approaches to meeting the goal, in ways that motivate and fulfil all parties, required open discussion and mutual understanding—but this was not always enough. For people with lived experience, the issues surrounding dementia can be traumatizing and present an urgent desire for change. Yet, researchers are typically trained and rewarded through the apparently slow system of grant funding and publications. To meet the aspirations of more immediate impact, knowledge translation roles have been created for people with lived experience, including working alongside researchers with policy makers for system-level advocacy (e.g., health policy change) [35], however, they are not commonly reported in research on dementia [36]. The EPLED Advisory Group expressed strong support for research that has potential for impact and, equally, frustration at the lack of uptake of the knowledge that already exists. Further, and more generally, a challenge to engagement in research continues to be the power imbalances that exist or may be perceived to exist between people with lived experience and researchers, attributed to factors such as differences in expertise and social status, economic hardship or poor health [37].

## Reflections on the importance of engagement

Through the first year, the EPLED Advisory Group members have discussed some of their motivations for engaging in CCNA research. Some common themes were:

### Challenging stigmas associated with dementia

One of the key motivations for the Advisory Group was the opportunity to challenge the stigmas associated with dementia—on the part of both researchers and people with lived experience. Through engagement in research, it is possible to highlight the contributions made by people with lived experience, challenge negative assumptions and create opportunities for voices to be heard. Stigma can be defined within the categories of public-stigma, or stigma that occurs within large social groups, such as within health care institutions, and self-stigma, where an individual may absorb or internalize stigma that is endorsed by the people around them [38]. Unfortunately, prevalent cultural depictions of dementia in film, television, literature, news, social media and language are often negative [39]. Stereotypical depictions of a person with dementia often include an old person with Alzheimer’s disease who behaves unpredictably—eventually losing all of their memory, and with it, their mind and identity [39]. These representations are often framed within the media as a someone who is not fully human (or is “othered”) and does not have a voice [39]. Public-stigma can create feelings of fear, anxiety and disgust toward people with dementia and self-stigma can cause feelings of frustration, anger, grief, loss of confidence, and depression [38]. Care partners, family, and friends of a person with dementia are also subject to the effects of stigma. Courtesy or associative stigma are terms coined to describe the discrimination and prejudice that people may experience because they are associated with individuals who belong to a stigmatised group. Affiliate stigma refers to the internalization of this stigma and can involve negative feelings that individuals develop toward themselves. As a result, the care partner, family member or friend may make less contact with the person who has dementia, conceal their association from the public, or generally pursue less social engagement [40]. Giving researchers an opportunity to get to know people with lived experience of dementia, including care partners, family and friends, would provide a deeper understanding of their unique experiences and needs. Through these relationships, it would be possible for researchers to see people living with dementia not just as subjects of research, but autonomous co-collaborators with diverse needs, thoughts and opinions. Engaging people living with dementia in research could help to challenge stigma about the perceived powerlessness of people living with dementia. Researchers, and the general public, have the potential to learn that people living with dementia are knowledgeable and possess the ability to actively and productively contribute to research.

### Creating meaning

Pursuing engagement in research has the potential to be very meaningful for both Advisory Group members and researchers [41]. Advisory Group members felt that by contributing to research and sharing their stories, it gives them an opportunity for their voice to be heard or that their experiences might help others. Developing collaborative relationships that are based on mutual respect, care and trust can colour this experience; these connections have the potential to be empowering. Research has shown that motivations for patients and caregivers to engage in health research include creating new relationships with others, helping others, learning more about research topics and having a voice in research [41]. Building a sense of community alongside researchers can also help to make research more accountable to the people who need it the most. However, more and better support is needed for people with lived experience of dementia who wish to become involved in research. For most, dementia is a devastating experience; virtual and physical spaces that are inclusive, accessible, non-stigmatizing and non-judgmental may help people with lived experience to feel that they are safe to express their thoughts and emotions and that their experience contributing to research can be meaningful.

### Contributing to research

Advisory Group members discussed their motivations to make research more relevant and impactful for people living with dementia and care partners. By sharing perspectives that are grounded in personal experience, people with lived experience of dementia could offer researchers new insights into issues and improve their understanding of community health needs [42–44]. Engagement may improve aspects of research studies, including feasibility, acceptability, rigor, relevance [45], and a meta-analysis reported that engagement activities likely improve enrollment in clinical trials [46]. Evaluating the impact of engagement on research will contribute to the emerging evidence base [47, 48]. Still, the Advisory Group also recognized the arguments for engagement extend beyond the benefit to research and includes discourse on the rights of people with lived experience [49].

### Identifying barriers to engagement

Working alongside Advisory Group members can also help researchers to identify barriers for people with lived experience of dementia in research. For example, when working with the Advisory Group, researchers were encouraged to consider ensuring accessibility of information (such as using lay-person language), adapting language or information (such as using visuals), developing trusting relationships, and respecting financial and time limitations [36, 49–52]. By identifying and addressing barriers to engagement, Advisory Group members hoped to enable other people living with dementia and care partners to become involved in research, including in roles as co-collaborators and knowledge users.

## Moving forward

EPLED and the Advisory Group found successes in the challenges of our first year together. Now in our second year, the Advisory Group also identified the need to address ongoing questions about engagement in research, including: involving individuals and groups typically excluded from research [37, 53] such as those from diverse racial, ethnic, 2SLGBTQIA + , language and Indigenous identities; building opportunities to engage in different types of research, including preclinical or biomedical research [54]; enabling patient led research [55]; and evaluating our program to improve EPLED and better support people with lived experience and CCNA researchers and trainees. We must also work to address barriers to involving people living with dementia; in the Advisory Group, care partners outnumber people living with dementia, and both offer important perspectives that we recognize are different [56–58]. Through moderating conversations and providing ongoing feedback and support, we will continue to prioritize many points of view by ensuring balanced opportunities to share thoughts. Finally, we must also challenge ourselves to find ways to work together to move research knowledge into action [59], including in initiatives to improve dementia care [60].

## Conclusion

The CCNA EPLED program and Advisory Group were created to support persons with dementia and care partners to be actively involved in the CCNA research process. The objective of this paper was to describe the activities of EPLED, as well as researchers and Advisory Group perceptions on the challenges and mitigation strategies, lessons learned and reflections on the importance of engagement from the first year of the EPLED Advisory Group. We hope these principles can be adapted for future research, including in other areas of research. People with lived experience of dementia can and should have roles in research beyond study subjects. The first year of the EPLED Advisory Group exemplified that people with lived experience of dementia are enthusiastic and effective co-collaborators in research. People with lived experience of dementia will impart new ideas, approaches and insights on dementia, create meaningful connections, and challenge stigmas associated with dementia. Building inclusive, respectful, and supportive relationships and spaces with individuals and communities—people living with dementia, care partners, researchers and research institutions—has the potential for important impact across these groups.

## Data Availability

Not applicable.

## Abbreviations

EPLED: Engagement of people with lived experience of dementia
CCNA: Canadian consortium of neurodegeneration in aging
SPOR: Strategy for patient oriented research
CIHR: Canadian institutes of health research
KITE: Knowledge, innovation, talent everywhere
KTE: Knowledge translation and exchange
